# A Meta-Analysis on the Long-Term Impact of Cytoreductive Surgery Plus HIPEC for Ovarian Cancer with Peritoneal Metastasis: Are We on the Right Path?

**DOI:** 10.3390/life16020335

**Published:** 2026-02-14

**Authors:** Dan Brebu, Mircea Șelaru, Ionut Flaviu Faur, Mihai Cosmin Burta, Ioana Adelina Faur, Amadeus Dobrescu, Ciprian Duță, Vlad Braicu, Andreea-Adriana Neamțu, Danau Răzvan

**Affiliations:** 1 IInd Surgery Clinic, Timisoara Emergency County Hospital, 300723 Timisoara, Romania; brebu.dan@umft.ro (D.B.); mihai.burta@umft.ro (M.C.B.); adelina.clim@umft.ro (I.A.F.); dobrescu.amadeus@umft.ro (A.D.); braicu.vlad@umft.ro (V.B.); 2 X Department of General Surgery, “Victor Babes” University of Medicine and Pharmacy Timisoara, 300041 Timisoara, Romania; duta.ciprian@umft.ro; 3 Department X, General Surgery II, Discipline of Surgical Emergencies, “Victor Babes” University of Medicine and Pharmacy Timisoara, Eftimie Murgu Square 2, 300041 Timisoara, Romania; 4 IIIrd Surgery Clinic, Timisoara Emergency County Hospital, 300723 Timisoara, Romania; 5 Doctoral School of Medicine, “Victor Babes” University of Medicine and Pharmacy Timisoara, Eftimie Murgu Square 2, 300041 Timisoara, Romania; 6 Pathology Department, Clinical County Emergency Hospital of Arad, Andrenyi Karoly Str, Nr. 2-4, 310037 Arad, Romania; andreea.neamtu@umft.ro; 7 Faculty of Pharmacy, “Victor Babes” University of Medicine and Pharmacy Timisoara, Eftimie Murgu Square 2, 300041 Timișoara, Romania; 8 Department of Urology, “Carol Davila” University of Medicine and Pharmacy, 8 Eroii Sanitari Blvd., 050474 Bucharest, Romania; razvan.danau@umfcd.ro; 9 Department of Urology, “Prof. Dr. Th. Burghele” Clinical Hospital, 20 Panduri Str., 050659 Bucharest, Romania

**Keywords:** HIPEC, cytoreductive surgery, ovarian cancer

## Abstract

Background: The benefit of adding hyperthermic intraperitoneal chemotherapy (HIPEC) to cytoreductive surgery (CRS) in ovarian cancer with peritoneal metastasis remains debated outside selected indications. We performed a systematic review and meta-analysis to quantify survival, perioperative morbidity, and completeness of cytoreduction using study-level data. Methods: PubMed/MEDLINE, Embase, and Web of Science were searched. Eligible English-language studies included ovarian cancer patients undergoing CRS plus HIPEC and reported at least one of the following: overall survival (OS), progression-free survival (PFS), Grade III–IV complications, or CC-0 rate. Random-effects meta-analyses were conducted using inverse-variance pooling. For HR outcomes, DerSimonian–Laird τ^2^ with Hartung–Knapp confidence intervals was applied. Proportions were pooled using logit transformation (PLOGIT) with random-effects models. Results: Twelve studies (*n* = 567) were included. Only two studies provided extractable HRs for OS and PFS (*n* = 217). CRS plus HIPEC was associated with improved OS (HR 0.68, 95% CI 0.52–0.90, *p* = 0.0023; I^2^ = 0%; prediction interval 0.14–3.34) and improved PFS (HR 0.70, 95% CI 0.31–1.57, *p* = 0.0007; I^2^ = 0%; prediction interval 0.18–2.66). Across 12 studies (*n* = 563), the pooled Grade III–IV complication rate was 0.18 (95% CI 0.14–0.22; I^2^ = 16.3%; prediction interval 0.12–0.26). In 10 studies (*n* = 385), the pooled CC-0 rate was 0.87 (95% CI 0.79–0.92; I^2^ = 46.7%; prediction interval 0.66–0.96). Conclusions: CRS plus HIPEC shows a favorable signal for OS and PFS in the limited HR-eligible evidence and appears feasible, with a pooled severe complication rate of ~18% and high CC-0 rates. Current data support HIPEC primarily as a targeted intensification strategy in carefully selected patients, while broader adoption requires additional randomized, context-specific evidence.

## 1. Introduction

Epithelial ovarian cancer remains the most lethal gynecologic malignancy worldwide. According to the latest World Health Organization GLOBOCAN 2022 estimates, the disease accounted for approximately 324,603 new cases and 206,956 deaths globally, underscoring its substantial oncologic burden [1]. Most patients present with advanced-stage disease characterized by extensive peritoneal dissemination and a high risk of relapse despite maximal cytoreductive surgery (CRS) and platinum-based chemotherapy [1,2]. This clinical trajectory highlights a persistent therapeutic challenge: achieving durable control of microscopic residual intraperitoneal disease even when macroscopically complete resection is obtained [3,4,5,6].

HIPEC, delivered intraoperatively immediately after CRS, is designed to intensify loco-regional treatment by combining high peritoneal drug exposure with heat-enhanced cytotoxicity and improved penetration into superficial tumor deposits [7,8,9,10,11]. The rationale is biologically compelling in ovarian cancer, where transcoelomic spread and peritoneal–tumor interactions drive disease persistence and recurrence. Yet the clinical signal remains debated, as the literature is heterogeneous in patient selection (primary/interval/recurrent), surgical intent, HIPEC technique, and drug regimens, and concerns persist regarding perioperative morbidity when HIPEC is added to complex multivisceral cytoreduction [7,8,12].

In this context, a synthesis that simultaneously addresses oncologic efficacy and perioperative safety is clinically necessary. We therefore conducted a systematic review and meta-analysis of CRS plus HIPEC in ovarian cancer with peritoneal metastasis, synthesizing overall survival and progression-free survival using extractable hazard ratios and quantifying perioperative morbidity and feasibility through pooled Grade III–IV complication rates and CC-0 rates.

## 2. Materials and Methods

This work was conducted as a systematic review and meta-analysis assessing oncologic outcomes and perioperative safety following cytoreductive surgery (CRS) combined with hyperthermic intraperitoneal chemotherapy (HIPEC) in ovarian cancer with peritoneal metastasis. The review was performed and reported according to the PRISMA 2020 statement [13].

Eligible studies enrolled adult patients with ovarian cancer and peritoneal metastasis treated with CRS followed by intraoperative HIPEC, irrespective of HIPEC technique (open or closed), perfusion parameters, or drug regimen. Studies were eligible if they reported at least one outcome of interest, including overall survival (OS), progression-free survival (PFS), severe postoperative morbidity, or completeness of cytoreduction. Randomized controlled trials, prospective cohorts, and retrospective cohorts were considered. Non-original publications (e.g., reviews, editorials), conference abstracts lacking extractable outcome data, duplicate or overlapping cohorts (for which the most complete or most recent dataset was retained), and studies not addressing ovarian cancer peritoneal metastasis treated with CRS + HIPEC were excluded.

### 2.1. Sources, Search Strategy and Study Selection

A systematic literature search was performed in PubMed/MEDLINE, Embase, and Web of Science (last searched on 10 December 2025). Reference lists of eligible full-text articles and relevant reviews were screened manually to identify additional reports. The search strategy combined controlled vocabulary and free-text terms related to ovarian cancer, HIPEC, and cytoreductive surgery. A representative PubMed strategy included terms for ovarian malignancy (e.g., “ovarian cancer”, “ovarian carcinoma”, “epithelial ovarian”), HIPEC (“hyperthermic intraperitoneal chemotherapy” OR HIPEC), and cytoreductive surgery (“cytoreductive surgery”, “debulking”, “peritonectomy”), combined with Boolean operators. 

All records retrieved from the electronic searches were imported into a reference management system and duplicates were removed. Titles and abstracts were screened, followed by full-text evaluation of potentially eligible reports. Screening and full-text assessment were conducted independently by two reviewers, and disagreements were resolved by consensus, with adjudication by a third reviewer when necessary.

**Figure 1:**
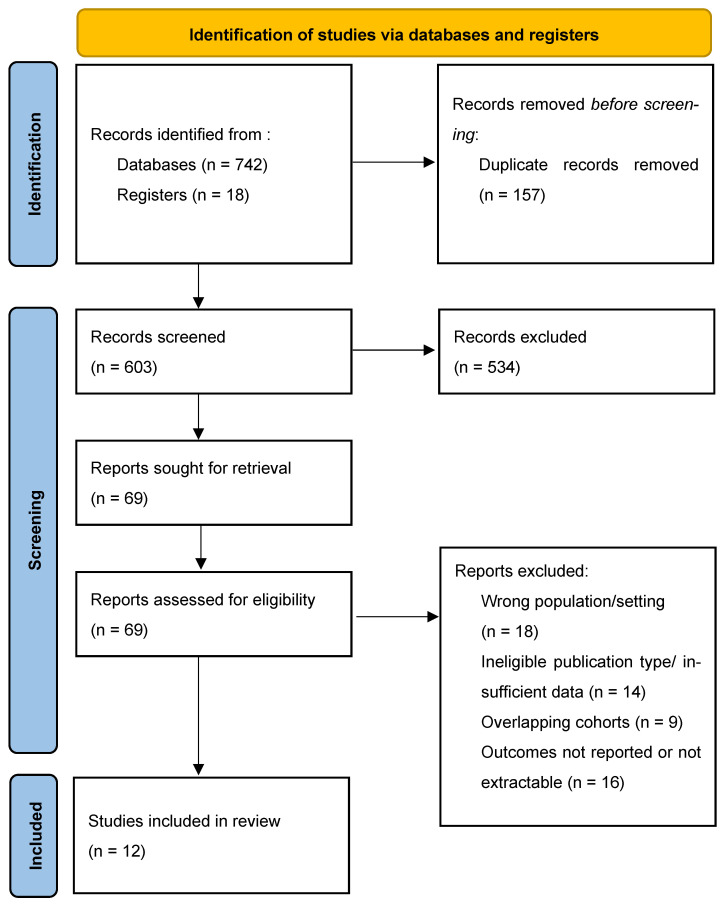
PRISMA flow diagram of selected studies.

The PRISMA-based study selection process is summarized as follows using placeholders that can be replaced if the original counts are recovered. A total of 742 records were identified through database searching and 18 additional records were identified through other sources, resulting in 603 records after duplicate removal. Title and abstract screening was performed for 603 records, of which 534 were excluded. Full texts were assessed for 69 articles, and 57 were excluded for predefined reasons, including wrong population or setting (*n* = 18), ineligible publication type or insufficient data (*n* = 14), overlapping cohorts (*n* = 9), and outcomes not reported or not extractable (*n* = 16). Twelve studies were included in the qualitative synthesis. Twelve studies contributed to quantitative synthesis for safety and feasibility outcomes, while only two studies provided extractable HRs for OS and two studies provided extractable HRs for PFS. The PRISMA flow diagram is presented in Figure 1.

### 2.2. Data Extraction and Outcome Definitions

Data extraction was performed independently by two reviewers using a standardized form. Extracted variables included study identifiers, design, clinical setting (primary, interval, or recurrent), surgical strategy, HIPEC technique and regimen, perfusion temperature and duration, sample size, follow-up duration, and disease burden metrics such as peritoneal cancer index (PCI) when available.

OS and PFS were preferentially extracted as hazard ratios (HRs) with corresponding 95% confidence intervals (CIs). When outcomes were reported without HRs, extraction was limited to data that were directly comparable or could be reliably transformed; otherwise, results were retained for narrative contextualization only. Perioperative morbidity was defined as severe postoperative complications corresponding to Grade III–IV severity based on Clavien–Dindo classification or equivalent grading systems as reported by each study. Completeness of cytoreduction was defined as CC-0 (no macroscopic residual disease) and extracted as a proportion. When CC-0 was reported as a percentage rather than an absolute count, the number of CC-0 events was derived from the reported sample size.

### 2.3. Risk of Bias and Statistical Analysis

Risk of bias was assessed at the study level using a design-appropriate, domain-based approach. Randomized controlled trials were assessed using a framework consistent with RoB 2, while non-randomized studies were assessed using a structured tool aligned with ROBINS-I principles. Each study was assigned an overall risk category based on the pattern and severity of potential biases. Any discrepancies between reviewers were resolved through consensus. 

All analyses were performed in R (R Foundation for Statistical Computing) using the meta and metafor packages. For time-to-event outcomes (OS and PFS), log-transformed HRs and standard errors were computed from reported HRs and 95% CIs. Random-effects meta-analysis was performed using inverse-variance weighting. Between-study variance (τ^2^) was estimated using the DerSimonian–Laird method, and random-effects confidence intervals were calculated using the Hartung–Knapp adjustment, which improves inference in settings with a limited number of studies. Statistical heterogeneity was quantified using τ^2^, I^2^, and Cochran’s Q test. Prediction intervals were computed to reflect the plausible range of effects in a new comparable study, when supported by the available evidence base.

For proportion outcomes, including Grade III–IV complication rate and CC-0 rate, random-effects meta-analysis of proportions was performed using a logit transformation with inverse-variance pooling. Between-study variance was estimated using DerSimonian–Laird and random-effects confidence intervals were calculated using Hartung–Knapp. Supportive analyses included leave-one-out analyses (metainf) and cumulative meta-analysis (metacum), provided to explore robustness and temporal stability of pooled estimates. Baujat plots were generated to visualize the influence of individual studies on heterogeneity and overall Q. Subgroup analyses were conducted only when each subgroup included at least two studies, to avoid unstable estimates and over-interpretation.

Publication bias and small-study effects were assessed only when at least 10 studies contributed to an outcome, consistent with methodological recommendations; therefore, these analyses were not performed for OS and PFS when fewer studies were available. Statistical significance was defined as a two-sided *p*-value < 0.05.

## 3. Results

### 3.1. Study Characteristics

A total of 12 studies were included, comprising 567 patients, reflecting a heterogeneous evidence base spanning randomized trials and non-randomized cohorts. Study design was predominantly non-randomized (retrospective cohorts: 6/12), while 3/12 were randomized controlled trials; the remainder consisted of prospective non-randomized or mixed-design cohorts. Regarding surgical context, most studies reported interval debulking surgery (IDS; 6/12) or secondary cytoreduction (SCR; 5/12), with primary debulking surgery (PDS) reported in 1/12 studies. HIPEC was delivered mainly via an open technique (10/12), with the closed technique reported in 2/12 studies. Cisplatin-based regimens predominated, most commonly cisplatin 100 mg/m^2^ (6/12). Study-level characteristics and extracted variables are summarized in Table 1.

Across studies, sample size ranged from 9 to 122 patients, supporting the presence of both small single-center experiences and larger multicenter/registry-level cohorts. Median follow-up ranged from 19.5 to 56.4 months (median 31.0 months). The burden of peritoneal disease, captured by PCI, varied substantially (mean PCI 8.5 to 19.7, median 14.0), consistent with clinically distinct case mixes across institutions and study settings. HIPEC delivery parameters were relatively consistent but not uniform: perfusion duration ranged from 60 to 90 min (median 90 min) and perfusate temperature ranged from 40.0 to 43.0 °C (median 42.1 °C) (Table 1).

### 3.2. Overall Survival

#### 3.2.1. Pooled Overall Survival

**Figure 2:**
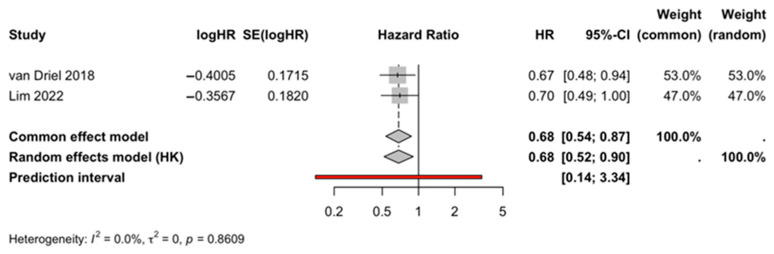
Overall survival (OS). Forest plot of the pooled hazard ratio (HR) for OS. Squares represent individual study estimates, with horizontal lines indicating 95% confidence intervals (CI). Diamond denotes the pooled hazard ratio (HR), and the vertical line indicates no effect [6,14].

Only two studies [6,14] reported OS as extractable hazard ratios (HRs) with 95% confidence intervals (CIs) in the provided dataset and were therefore eligible for OS meta-analysis. The pooled analysis showed significantly improved OS associated with CRS plus HIPEC (pooled HR = 0.684, 95% CI 0.536–0.874, *p* = 0.0023; I^2^ = 0%, τ^2^ = 0; heterogeneity Q-test *p* = 0.861) (Figure 2, Table 2). Consistent with I^2^ = 0% and τ^2^ = 0, the pooled estimate suggested minimal between-study statistical heterogeneity among the eligible studies for OS; however, this should be interpreted cautiously given the limited number of contributing studies.

Prediction intervals were computed in the model framework to reflect the range of effects that might be expected in a future comparable study. While informative, the prediction interval is inherently constrained by the small number of included studies and should be interpreted as supportive rather than definitive in this context.

#### 3.2.2. Sensitivity and Supporting Analyses (OS)

Leave-one-out sensitivity analysis was necessarily limited by the inclusion of only two studies; nevertheless, it is provided for completeness to document the stability of the pooled estimate under sequential study exclusion. Cumulative meta-analysis and Baujat diagnostics were generated as supportive analyses. Given the small number of studies, these diagnostics should be viewed primarily as transparency tools rather than as strong evidence for or against the influence of any single study.

Formal small-study effects testing (e.g., Egger regression test) and trim-and-fill correction were not statistically appropriate for OS, as fewer than 10 studies contributed to the time-to-event synthesis.

### 3.3. Progression-Free Survival

#### 3.3.1. Pooled Progression-Free Survival

**Figure 3:**
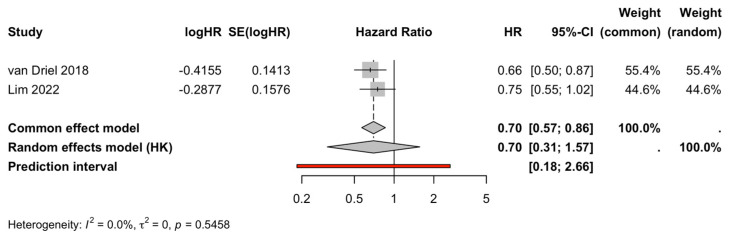
Progression-free survival (PFS). Forest plot of the pooled hazard ratio (HR). Squares represent individual study estimates, with horizontal lines indicating 95% confidence intervals (CI). Diamond denotes the pooled hazard ratio (HR), and the vertical line indicates no effect [6,14].

Similarly, only two studies [6,14] provided extractable PFS hazard ratios (HRs) with 95% confidence intervals (CIs) and were therefore eligible for meta-analysis. The pooled estimate favored CRS plus HIPEC for progression-free survival (pooled HR = 0.70, 95% CI 0.31–1.57; I^2^ = 0%, τ^2^ = 0; heterogeneity Q-test p = 0.546) (Figure 3, Table 2). However, the wide confidence interval reflects the limited number of eligible studies and underscores the need for cautious interpretation rather than definitive inference regarding clinical benefit.

#### 3.3.2. Sensitivity and Supporting Analyses (PFS)

Sensitivity checks were performed to explore the stability of the pooled PFS estimate. The direction and magnitude of the effect were broadly maintained when analyses were re-run after omitting individual studies and when evidence was examined cumulatively, suggesting no obvious single-study dependence. Formal assessment of small-study effects (e.g., funnel plot-based methods) was not emphasized given the limited number of studies contributing extractable PFS hazard ratios.

### 3.4. Grade III–IV Postoperative Complications

**Figure 4:**
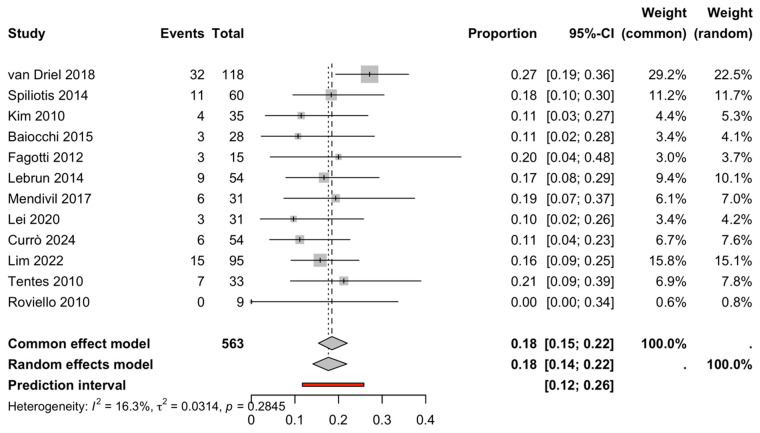
Severe postoperative morbidity (Grade III–IV). Forest plot of the pooled Grade III–IV postoperative complication rate across included studies. Squares represent study proportions with 95% confidence intervals (CI). The diamond indicates the pooled estimate, and the vertical line represents the overall effect [4,6,8,9,11,12,14,15,16,17,18,19].

All 12 studies provided extractable Grade III–IV complication counts (events/total), enabling synthesis of the severe complication rate. The pooled Grade III–IV complication rate was 17.7% (95% CI 14.3–21.7%), with low-to-moderate heterogeneity (I^2^ = 16.3%, τ^2^ = 0.031 on the logit scale; Q-test *p* = 0.285) (Figure 4, Table 2). The corresponding prediction interval suggested that the expected severe complication rate in a new comparable study would plausibly fall within 12.3–24.8%, indicating clinically meaningful variability across settings despite relatively modest statistical heterogeneity.

Given the study-level nature of the extracted complication data, this synthesis represents an event-rate meta-analysis rather than a direct comparative risk estimate (HIPEC vs. non-HIPEC). Where comparator arms exist in original publications but were not encoded as two-arm data in the current dataset, comparative effect measures (RR/OR) could not be computed within the present workflow.

### 3.5. Perioperative Morbidity and Safety

Perioperative safety was primarily evaluated through study-level reporting of severe postoperative complications (Grade III–IV). All 12 studies provided extractable Grade III–IV complication counts (events/total), enabling synthesis of the pooled severe complication rate. The random-effects proportion meta-analysis yielded an estimated Grade III–IV complication rate of 17.7% (95% CI 14.3–21.7%) with low-to-moderate heterogeneity (I^2^ = 16.3%, Q-test *p* = 0.285) (Figure 4, Table 2). The corresponding prediction interval (12.3–24.8%) suggests that, in a new comparable setting, the expected severe complication rate may vary within a clinically relevant range, reflecting differences in patient selection, operative complexity, perioperative care pathways, and institutional experience.

In addition to morbidity, completeness of cytoreduction represents a key procedural endpoint and an indirect marker of operative feasibility within the studied populations.

**Figure 5:**
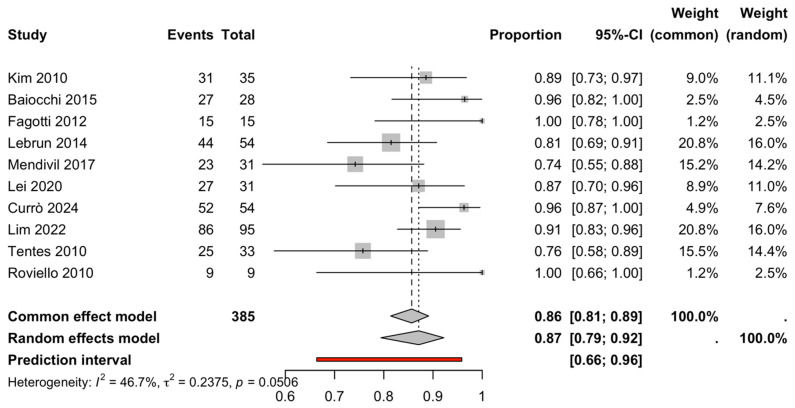
Completeness of cytoreduction (CC-0 rate). Forest plot of the pooled CC-0 rate. Squares represent study proportions with 95% confidence intervals (CI). The diamond indicates the pooled estimate, and the vertical line represents the overall effect [4,6,9,11,12,15,16,17,18,19].

Ten studies reported CC-0 alongside sample size, allowing meta-analysis of the CC-0 rate. The pooled CC-0 rate was high (87.1%, 95% CI 80.8–91.5%) with moderate heterogeneity (I^2^ = 46.7%, Q-test *p* = 0.051) (Figure 5, Table 2), and a wider prediction interval (69.9–95.1%), consistent with variability in disease burden (e.g., PCI), surgical intent (IDS vs. SCR), and technical approach across centers.

Importantly, the present perioperative morbidity synthesis reflects an event-rate meta-analysis based on aggregated study-level reporting. As two-arm comparative complication data (HIPEC vs. non-HIPEC) were not encoded uniformly in the current dataset, comparative effect estimates (RR/OR) could not be generated within this workflow. Nevertheless, the pooled severe complication rate and CC-0 rate provide a clinically meaningful overview of perioperative morbidity and feasibility in published CRS + HIPEC series, supporting interpretability of the survival analyses in the context of operative safety.

### 3.6. Completeness of Cytoreduction (CC-0 Rate)

Ten studies reported CC-0 alongside sample size, allowing synthesis of the CC-0 rate. The pooled CC-0 rate was 87.1% (95% CI 80.8–91.5%) (Figure 5, Table 2), with moderate heterogeneity (I^2^ = 46.7%, τ^2^ = 0.237 on the logit scale; Q-test *p* = 0.051). The prediction interval was wider (69.9–95.1%), consistent with differences in baseline disease burden (PCI), institutional surgical volume, selection criteria, and cytoreductive strategy across studies.

### 3.7. Additional Descriptive Outputs and Data Transparency

To strengthen reproducibility, we compiled a complete study-level extraction dataset covering all variables used in the analyses and generated descriptive summaries to characterize between-study variability and data completeness. These outputs were used to confirm internal consistency of extracted fields (e.g., ranges of follow-up, PCI, HIPEC temperature and duration) and to document the extent of missingness for each variable, thereby clarifying which study-level covariates could be meaningfully explored and which could not.

## 4. Discussion

### 4.1. Context and Timing of HIPEC in Primary Ovarian Cancer

Our comprehensive analysis indicates that combining cytoreductive surgery with HIPEC offers a promising strategy to improve long-term outcomes in advanced ovarian cancer with peritoneal metastasis, especially when used during interval debulking surgery (IDS) for stage III disease. In this setting, randomized trials have shown a significant survival benefit—for example, the Dutch OVHIPEC-1 trial reported a median overall survival of 45.7 months with HIPEC vs. 33.9 months without (a ~12-month increase) [14,20,21]. Longer-term follow-up has confirmed a persistent survival advantage with HIPEC at IDS [21]. Pooled analyses and meta-reviews support a clinically meaningful reduction in mortality risk when HIPEC is added in appropriately selected settings, particularly after NACT/IDS [22,23]. Accordingly, a growing body of evidence has led major centers and guidelines to cautiously endorse HIPEC in specific scenarios—notably, NCCN includes HIPEC with cisplatin as an option in the IDS context for stage III disease after neoadjuvant chemotherapy [24].

One clear theme is that the context in which HIPEC is used matters greatly. In primary advanced ovarian cancer, when HIPEC is applied at IDS after neoadjuvant chemotherapy (NACT), randomized evidence and RCT-focused meta-analyses show the most consistent benefit [14,21,22]. Biologically, this scenario is plausible, and clinically, it avoids treatment disruption because chemotherapy is delivered intra-operatively without requiring an additional treatment phase. More broadly, delays in initiating or resuming systemic chemotherapy after debulking have been associated with worse outcomes in ovarian cancer, supporting strategies that do not introduce avoidable postoperative delays [25,26]. Indeed, OVHIPEC-1 integrated cisplatin HIPEC during IDS without delaying adjuvant chemotherapy and demonstrated improved progression-free and overall survival [14,20,21,24]. Additional randomized data also suggest that any survival signal is concentrated in the IDS-after-NACT subgroup rather than in primary surgery overall [6]. Observational data on recurrence patterns after CRS + HIPEC are also consistent with a potential peritoneal control effect, although these designs remain hypothesis-generating [27].

By contrast, using HIPEC at the time of primary debulking surgery (PDS) (i.e., upfront surgery before any chemotherapy) has not yet shown clear benefit. Notably, OVHIPEC-2 is specifically designed to address HIPEC at primary cytoreduction prospectively [20]. Retrospective studies in the PDS setting have been mixed, and recent meta-analyses have not demonstrated consistent survival benefit for HIPEC at upfront primary surgery compared with surgery alone [22,23]. Some expert commentaries have therefore cautioned against broad adoption outside trials and emphasized that current evidence does not redefine standard first-line care universally [28]. Thus, the “right path” for HIPEC in newly diagnosed disease appears to be its use at interval surgery (after NACT) rather than at upfront primary debulking until primary-surgery RCT data mature [20].

### 4.2. HIPEC in Recurrent Ovarian Cancer

For platinum-sensitive recurrent ovarian cancer, the role of HIPEC is more nuanced. The divergent findings of the French CHIPOR trial versus the Italian HORSE (MITO-18) trial emphasize that patient selection and trial context are critical. CHIPOR (Phase III) demonstrated a statistically significant overall survival benefit with HIPEC added to surgery for first relapse [29]. In contrast, HORSE/MITO-18 did not show a progression-free survival advantage with HIPEC added to (near-)complete secondary cytoreduction, though post-recurrence survival estimates were numerically higher in the HIPEC arm [30]. These findings suggest that HIPEC in recurrence may benefit only a subset of patients under specific clinical conditions [29,30,31].

A key difference between trials was the systemic-therapy context and timing of surgery. CHIPOR’s design incorporated platinum-based chemotherapy before surgical allocation, effectively enriching for platinum-sensitive, responsive disease before adding HIPEC [29]. HORSE randomized at surgery in patients undergoing secondary cytoreduction without neoadjuvant chemotherapy for relapse and found no PFS benefit [30]. Differences in maintenance-era systemic therapy and baseline prognosis likely contributed to variability in effect size across studies, supporting a conservative interpretation that HIPEC’s incremental value in recurrence is context-dependent rather than universal.

Other trial design differences further complicate comparisons, including sample size, endpoints, and selection criteria. In this regard, meta-analytic synthesis suggests that HIPEC effects can vary with the timing of systemic chemotherapy exposure and treatment setting, reinforcing the centrality of careful indication definition rather than “one-size-fits-all” use [22,32].

Taken together, current evidence suggests that HIPEC in recurrence is most plausibly beneficial in carefully selected patients—particularly those with platinum-sensitive first relapse and a realistic prospect of complete secondary cytoreduction—while routine use across broader recurrent populations remains unsupported. Supporting randomized data in recurrence include both CHIPOR and earlier trials, such as the Spiliotis phase III study [8,29].

Future work should refine selection criteria, potentially incorporating biomarkers. In OVHIPEC translational subgroup analyses, benefit signals differed across HRD/BRCA strata, raising the hypothesis that HIPEC’s incremental value may depend on underlying DNA repair phenotype [33].

### 4.3. Safety and Feasibility of HIPEC

Our analysis supports that HIPEC, when delivered in experienced programs, is feasible and does not necessarily translate into prohibitive perioperative mortality. In OVHIPEC-1, the addition of HIPEC did not increase the rate of severe postoperative adverse events compared with surgery alone [14,21]. In CHIPOR, grade ≥ 3 events were more frequent with HIPEC, but 60-day postoperative mortality was not higher and events were largely driven by metabolic/laboratory abnormalities as reported in the trial [29]. RCT-level meta-analysis also supports broadly comparable rates of grade ≥ 3 adverse events between HIPEC and control across randomized evidence [22].

Quality-of-life (QoL) data are also reassuring. In the OVHIPEC HRQoL analysis, adding HIPEC to interval CRS did not negatively impact HRQoL trajectories compared with surgery alone [20,33].

### 4.4. Current Adoption and Outlook

As of 2025, adoption varies internationally, reflecting both the positive IDS evidence and the desire for confirmatory data in other settings. NCCN Guidelines Insights include HIPEC as a consideration in the IDS context, while emphasizing maximal cytoreduction and appropriate patient selection [24]. In parallel, prospective institutional experiences have explored expanding IDS + HIPEC beyond the strict OVHIPEC-1 population, while keeping selection constraints and reporting perioperative outcomes [34].

That said, skepticism has persisted, especially regarding extrapolation to upfront primary surgery and heterogeneous recurrent settings. Expert commentary has emphasized that evidence should not be generalized beyond the settings supported by robust data, pending further randomized trials [20,28]. More recent meta-analyses continue to converge on the same practical conclusion: the most consistent benefit is in the IDS-after-NACT context, while survival benefits at upfront PDS or broadly in recurrence are not consistently demonstrated [2,6,22,23,25].

### 4.5. Clinical Implications

From a practical perspective, the current evidence supports HIPEC primarily as a targeted intensification strategy in carefully selected patients, particularly those undergoing interval debulking surgery after neoadjuvant chemotherapy, where the survival signal appears most consistent [11,16,20]. Routine implementation outside experienced centers or in unselected patient populations is not supported by the available data [4,12]. Therefore, appropriate patient selection, institutional expertise, and multidisciplinary evaluation remain essential to safely translate HIPEC into meaningful oncologic benefit while avoiding unnecessary procedural morbidity [30,32,33,34].

## 5. Limitations

We acknowledge limitations in both the underlying evidence and our synthesis. Included studies remain heterogeneous in design, patient selection, HIPEC technique, and systemic therapy context, and non-randomized data are intrinsically vulnerable to selection bias and confounding. Even within randomized evidence, differences in setting (primary vs. interval vs. recurrent), eligibility criteria, and endpoints complicate cross-trial comparisons. QoL and patient-reported outcomes remain inconsistently captured, though available randomized data suggest no major HRQoL decrement attributable to HIPEC in the IDS setting [20,24,35]. Ongoing trials—particularly those evaluating HIPEC at primary cytoreduction—will be essential for defining whether HIPEC’s role can responsibly expand beyond its current evidence-supported niche [20].

## 6. Conclusions

In the current evidence base, CRS plus HIPEC shows its most consistent clinical value when delivered at interval debulking after neoadjuvant chemotherapy for stage III ovarian cancer, where randomized data support a meaningful survival benefit without prohibitive perioperative mortality. Outside this setting—particularly at upfront primary debulking and across unselected recurrent disease—the evidence is heterogeneous and does not justify routine implementation. In platinum-sensitive first relapse, HIPEC may be beneficial in highly selected patients undergoing complete secondary cytoreduction, but this remains context-dependent. Overall, HIPEC should be considered a targeted intensification strategy performed in experienced centers, while ongoing trials and biomarker-informed selection are essential to define its precise role and avoid overextension beyond evidence-supported indications.

## Figures and Tables

**Table 1:** Study characteristics.

Study ID	Country	Study Type	Sample Size	Surgery Type	Hipec Regimen	Technique	Duration (Min)	Temperature (°C)	Follow-Up (Months)	PCI (Mean)
van Driel, 2018 [14]	The Netherlands	RCT	122	IDS	Cisplatin 100 mg/m^2^	Open	90	40	56.4	NA
Spiliotis, 2014 [8]	Greece	RCT	60	SCR	Cisplatin 100 mg/m^2^	Open	90	42.5	39	15
Kim, 2010 [15]	South Korea	Retrospective Cohort	35	SCR	Cisplatin 75 + Paclitaxel 175 mg/m^2^	Open	90	42.25	24.5	8.5
Baiocchi, 2015 [11]	Brazil	Prospective Cohort	28	SCR	Cisplatin 75 + Doxorubicin 15 mg/m^2^	Closed	90	41.5	26.6	15.5
Fagotti, 2012 [9]	Italy	Prospective Pilot	15	IDS	Cisplatin 100 mg/m^2^	Open	90	41.5	30	11
Lebrun, 2014 [4]	France	Retrospective Cohort	54	SCR	Cisplatin 75 + Doxorubicin 15 mg/m^2^	Open	60	42	38.5	14
Mendivil, 2017 [16]	USA	Retrospective Cohort	31	IDS	Cisplatin 100 mg/m^2^	Open	90	41.5	19.5	NA
Lei, 2020 [17]	China	Retrospective Cohort	31	IDS	Cisplatin 70 mg/m^2^	Open	60	43	24	19.7
Currò, 2024 [12]	Italy	Retrospective Cohort	54	IDS	Cisplatin 100 mg/m^2^	Open	90	42.5	28.6	12.5
Lim, 2022 [6]	South Korea	RCT	95	IDS	Cisplatin 75 mg/m^2^	Open	90	42.5	39.5	14
Tentes, 2010 [18]	Greece	Retrospective Cohort	33	SCR	Cisplatin 100 mg/m^2^	Open	90	42.5	32	NA
Roviello, 2010 [19]	Italy	Prospective Case Series	9	PDS	Cisplatin 100 + Paclitaxel 60 mg/m^2^	Closed	90	41.75	48	14

Abbreviations: CC-0, no macroscopic residual disease (complete cytoreduction); HIPEC, hyperthermic intraperitoneal chemotherapy; IDS, interval debulking surgery; NA, not available/not reported; PCI, Peritoneal Cancer Index; PDS, primary debulking surgery; RCT, randomized controlled trial; SCR, secondary cytoreductive surgery.

**Table 2:** Summary of pooled effects (random-effects meta-analysis).

Outcome	Studies (k)	Total Patients (*n*)	Effect Measure	Model (Τ^2^/Ci Method)	Pooled Effect (Random)	*p*-Value	I^2^ (%)	τ^2^	Q-Test *p*-Value	Prediction Interval
Overall Survival (Os)	2	217	HR	DL/Hartung–Knapp	0.68 (0.52–0.90)	0.0023	0.0	0	0.8609	0.14–3.34
Progression-Free Survival (Pfs)	2	217	HR	DL/Hartung–Knapp	0.70 (0.31–1.57)	0.0007	0.0	0	0.5458	0.18–2.66
Grade Iii–Iv Complications	12	563	Proportion	DL/Hartung–Knapp (PLOGIT)	0.18 (0.14–0.22)	—	16.3	0.0314	0.2845	0.12–0.26
Cc-0 Rate	10	385	Proportion	DL/Hartung–Knapp (PLOGIT)	0.87 (0.79–0.92)	—	46.7	0.2375	0.0506	0.66–0.96

Abbreviations: CI, confidence interval; DL, DerSimonian–Laird; HR, hazard ratio; HK, Hartung–Knapp; PLOGIT, logit-transformed proportion; τ^2^, between-study variance; I^2^, heterogeneity statistic.

## Data Availability

No new data were created or analyzed in this study.
